# The COP9 signalosome subunit CSN1 regulates glioblastoma stem cell growth

**DOI:** 10.1371/journal.pone.0358568

**Published:** 2026-09-17

**Authors:** Samir Assaf, Kyle Heemskerk, Xiaoguang Hao, H. Artee Luchman, Samuel Weiss

**Affiliations:** 1 Arnie Charbonneau Cancer Institute, Cumming School of Medicine, University of Calgary, Calgary, Alberta, Canada; 2 Hotchkiss Brain Institute, Cumming School of Medicine, University of Calgary, Calgary, Alberta, Canada; 3 Department of Cell Biology and Anatomy, University of Calgary, Calgary, Alberta, Canada; Keio University School of Medicine Graduate School of Medicine: Keio Gijuku Daigaku Igakubu Daigakuin Igaku Kenkyuka, JAPAN

## Abstract

The COP9 signalosome is composed of 8 subunits (CSN1-CSN8) and plays a key role in regulating the activity of the ubiquitin-proteasome system by modulating Cullin-RING ubiquitin ligases. While the function of the COP9 signalosome in regulating protein homeostasis is well established, little is known about its role in glioblastoma. Here, using patient-derived glioblastoma stem cell lines, we identified CSN1 as a crucial COP9 signalosome subunit for *in vitro* growth. CSN1 knockout was further found to significantly extend survival in orthotopic glioblastoma xenograft models. Transcriptomic analysis revealed that CSN1 knockout disrupted transcriptional programs involved in cell cycle progression and activated apoptotic pathways. Molecular analysis further determined that CSN1 is required for COP9 signalosome complex assembly and regulates AP-1 activation via JNK1/2/3 and c-Jun phosphorylation. These results highlight the essentiality of CSN1 for glioblastoma stem cell growth and provide insights into the cellular mechanisms controlled by CSN1 and the COP9 signalosome.

## Introduction

Proteostasis is a vital system that ensures the balance of regulatory proteins in the cell is maintained, ultimately controlling critical functions and responses to external stimuli [1]. One of the key mechanisms involved in regulating proteostasis is the ubiquitin-proteasome system (UPS), a multi-component system that relies on the E1-E2-E3 ubiquitin conjugation cascade to selectively mark unwanted proteins for degradation by the proteasome [1,2]. The largest family of E3 complexes are the Cullin-RING ubiquitin ligases, where a Cullin acts as a scaffold for a RING to transfer ubiquitin from a ubiquitin-carrying E2 to a substrate protein [2]. However, Cullins require NEDD8 neddylation to maintain the flexibility needed to bring the RING-E2 closer to the substrate protein, thereby enabling efficient transfer of ubiquitin to the substrate protein [2].

A critical modulator of the UPS process is the highly conserved hetero-octameric protein complex, the COP9 signalosome [3]. The COP9 signalosome acts as an isopeptidase for NEDD8, fine-tuning Cullin-RING ubiquitin ligase activity and substrate specificity for ubiquitination and subsequent degradation [3]. A growing body of evidence indicates a COP9 subunit-specific involvement in regulating either oncogenic or tumor suppressor programs in a cancer-dependent manner (reviewed in [4]). For example, CSN1 was found to contribute to hepatocellular carcinoma migration and proliferation by upregulating cyclinA2 expression [5]. However, CSN1 was also suggested to act as a tumor suppressor in penile squamous cell carcinoma as CSN1 mutations disrupted miRNA-mediated gene silencing, contributing to disease development [6]. The variability in cellular programs regulated by the COP9 signalosome in different cancers highlights the need to unbiasedly dissect the involvement, and importance, of each subunit in different cancer types.

CSN6 is the only COP9 subunit investigated in glioblastoma, where it was shown to drive tumor cell proliferation by stabilizing the mitogen receptor EGFR [7]. However, despite the therapeutic interest in targeting the UPS in glioblastoma, the contribution of the other COP9 signalosome subunits within this process remains poorly understood. In this study, we investigated the essentiality of COP9 signalosome subunits for glioblastoma stem cell (GSC) growth *in vitro*. We show that, alongside CSN6 whose role has been previously investigated [7], CSN1 is also essential for GSC growth *in vitro.* We demonstrate that CSN1 regulates transcriptional profiles associated with the activation of the AP-1 transcription factor and cell cycle progression. Furthermore, we provide the first evidence that CSN1 regulates GSC-driven tumor growth.

## Results

### The COP9 signalosome subunit, CSN1, is essential in GSCs

**Fig 1:**
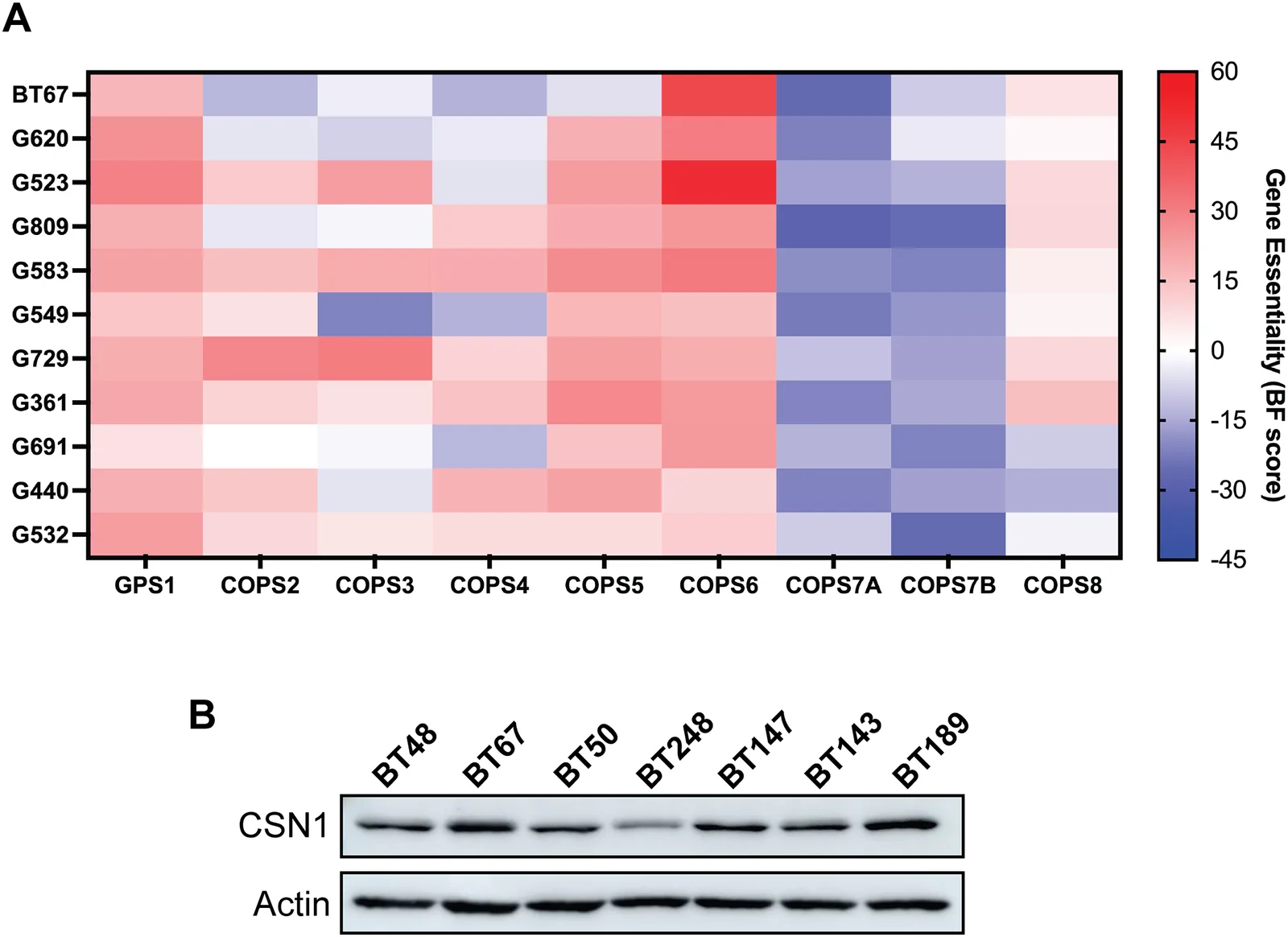
CSN1 and CSN6 are the most essential COP9 subunits in GSCs. **(A)** Heatmap displaying the Bayes factor essentiality score of genes encoding COP9 signalosome complex subunits in 11 GSC cultures. **(B)** CSN1 protein expression in 7 patient-derived GSC cultures.

The COP9 signalosome is composed of 8 subunits (CSN1, CSN2, CSN3, CSN4, CSN5, CSN6, CSN7A/7B, and CSN8) [8] which are encoded by *GPS1, COPS2, COPS3, COPS4, COPS5, COPS6, COPS7A/7B,* and *COPS8*, respectively. To identify the most critical subunit in GSCs, we analyzed a previously published genome-wide essentiality screen that encompassed 11 GSCs [9]. Interestingly, knockout of only two COP9 signalosome subunit genes, *GPS1* and *COPS6*, which encode CSN1 and CSN6, respectively, conferred a fitness defect in all 11 GSC cultures (Fig 1A). Given that the role of CSN6 in glioblastoma has been previously investigated [7], our current study focused on elucidating the contributions of CSN1 to the disease. CSN1 expression was subsequently assessed across a diverse panel of GSC cultures to confirm its broad expression (Fig 1B).

### Loss of CSN1 stunts GSC growth and stemness *in vitro* and prolongs survival in orthotopic glioblastoma xenograft models

**Fig 2:**
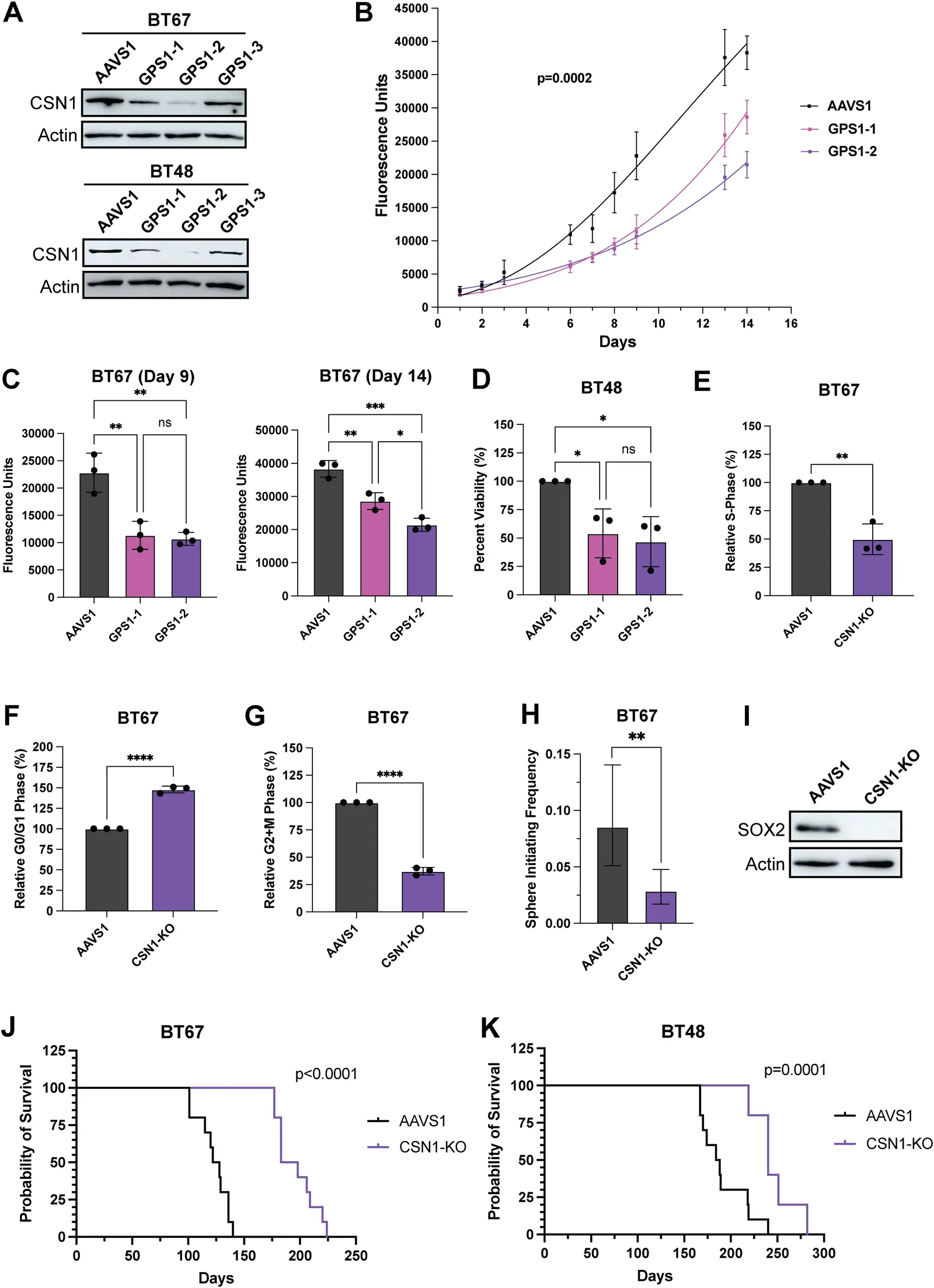
CSN1 promotes GSC growth and tumorigenesis. **(A)** Western blot validation of CSN1 knockout in BT67 (n = 3) and BT48 (n = 3). **(B)** Growth curves of AAVS1, GPS1–1, and GPS1–2 BT67 GSC cultures over 14 days. Gompertz growth model line of best fit; data represented as mean + /- SD, n = 3. **(C)** AAVS1, GPS1–1, and GPS1–2 BT67 GSC culture viability at 9 and 14 days. Data points were extracted from the growth curve in (B). ANOVA (Tukey’s test) at 95% confidence intervals, ns = not significant, *p < 0.05, **p < 0.01, ***p < 0.001; data represented as mean + /- SD, n = 3. **(D)** Viability of AAVS1, GPS1–1, and GPS1–2 BT48 GSC cultures after 7 days. ANOVA (Tukey’s test) at 95% confidence intervals, ns = not significant, *p < 0.05; data represented as mean + /- SD, n = 3. **(E)** Flow cytometry EdU incorporation assay highlighting the percentage of CSN1-KO GSCs in S-phase relative to AAVS1. Unpaired two-tailed t-test, **p < 0.01, data represented as mean ± SD, n = 3. **(F)** Flow cytometry EdU incorporation assay highlighting the percentage of CSN1-KO GSCs in G0/G1 phase relative to AAVS1. Unpaired two-tailed t-test, ****p < 0.0001, data represented as mean ± SD, n = 3. **(G)** Flow cytometry EdU incorporation assay highlighting the percentage of CSN1-KO GSCs in G2 + M phase relative to AAVS1. Unpaired two-tailed t-test, ****p < 0.0001, data represented as mean ± SD, n = 3. **(H)** Limiting dilution assay of BT67 AAVS1 and CSN1-KO GSCs. Chi-squared test, **p < 0.01; data represented as mean + /- upper/lower limit, n = 6. **(I)** Western blot analysis of SOX2 expression in AAVS1 and CSN1-KO BT67 GSCs (n = 3). **(J)** Kaplan-Meier survival curve of AAVS1 (n = 10) and CSN1-KO BT67 GSCs (n = 10). p < 0.0001; Log-rank test. **(K)** Kaplan-Meier survival curve of AAVS1 (n = 10) and CSN1-KO BT48 GSCs (n = 10). p < 0.0001; Log-rank test**.**

To elucidate the functional contributions of CSN1 in GSCs, we performed CRISPR-Cas9 mediated knockout of *GPS1*, which encodes CSN1, with three gRNAs in two different patient-derived GSC cultures (Fig 2A). This generated stable GSCs with ~20% residual CSN1 expression (GPS1–1) and a near complete knockout of CSN1 (GPS1–2), relative to the AAVS1 control (Fig 2A). We investigated the effect of CSN1 loss on GSC growth by tracking the growth rate of GPS1–1 and GPS1–2 compared to AAVS1 over a 14-day time course (Fig 2B). Results showed that reduced CSN1 expression due to CRISPR-Cas9 targeting significantly inhibited GSC growth over time (Fig 2B-D). Since GSCs targeted with the second *GPS1* gRNA (GPS1–2) displayed the highest CSN1 knockout efficiency, we utilized them for all remaining experiments in the study and referred to them as CSN1-KO. We then validated the effect of CSN1 on GSC growth by performing EdU flow cytometric analysis. This revealed that CSN1-KO disrupts normal cell cycle progression, displaying a reduced percentage of cells in S and G2 + M phases while accumulating cells in G0/G1 phase relative to the AAVS1 control (Fig 2E-G). We next performed a limiting dilution assay on AAVS1 and CSN1-KO GSCs to determine whether CSN1 regulates GSC stemness. This revealed that CSN1-KO significantly reduced GSC sphere-initiating frequency, correlating with a dramatic reduction in the GSC stemness marker, SOX2 (Fig 2H-I). To determine whether the *in vitro* growth and stemness defects translated *in vivo,* we orthotopically xenografted two independent AAVS1 and CSN1-KO GSC cultures into SCID mice. Strikingly, we observed that mice xenografted with CSN1-KO GSCs exhibited a significant survival advantage relative to AAVS1 GSCs (Fig 2J-K).

### CSN1 regulates cell cycle and apoptotic transcriptional programs in GSCs

**Fig 3:**
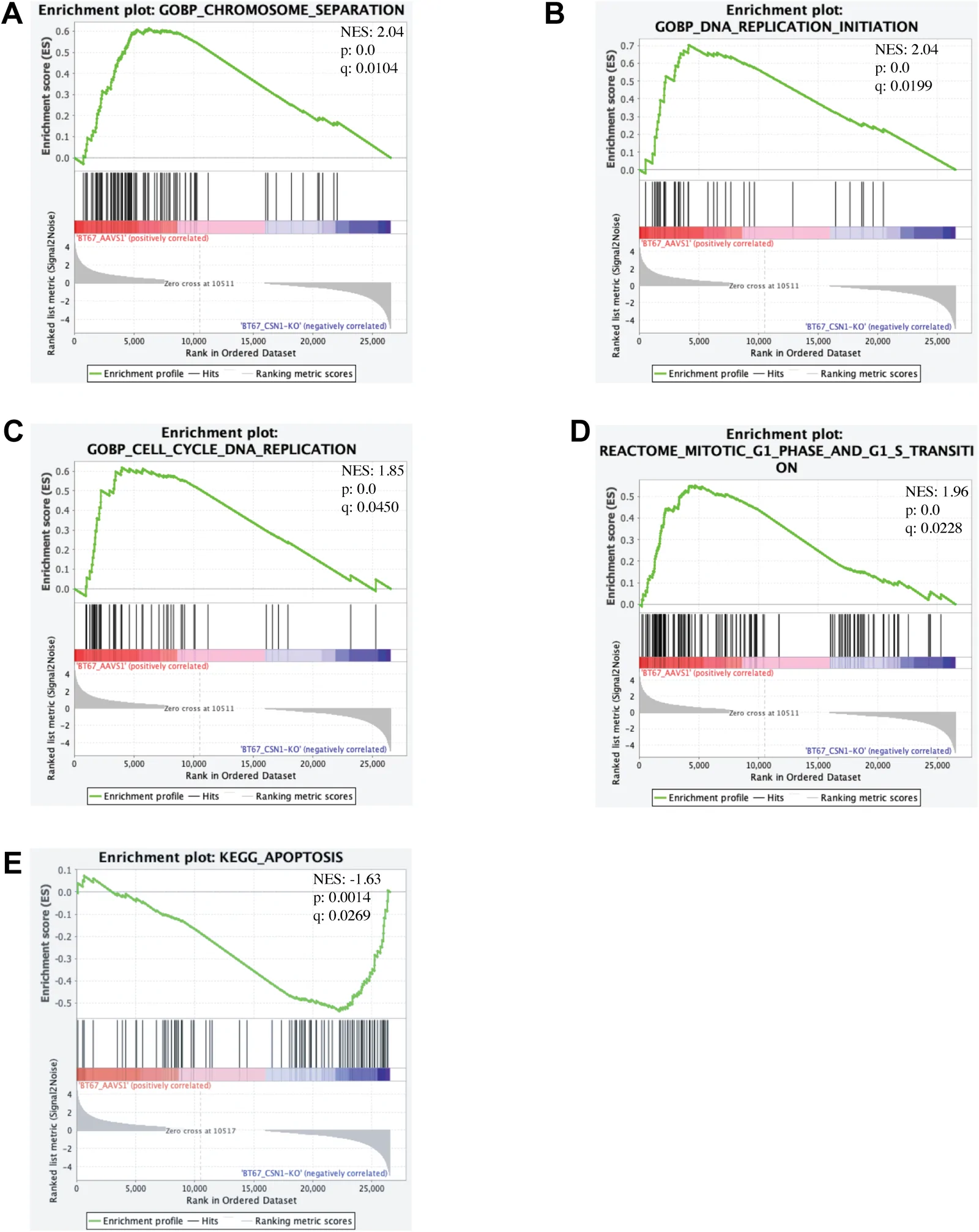
CSN1 regulates transcriptional programs associated with cell cycle progression and apoptosis. **(A-C)** GSEA biological processes analysis showing enriched cell cycle processes in AAVS1 versus CSN1-KO GSCs. NES represents the normalized enrichment score, p-value represents the hypergeometric test and q-value represents the false-discovery corrected p-value (n = 3). **(D)** GSEA reactome pathway analysis showing increased G1-S cell cycle transition in AAVS1 versus CSN1-KO GSCs. NES represents the normalized enrichment score, p-value represents the hypergeometric test and q-value represents the false-discovery corrected p-value (n = 3). **(E)** GSEA KEGG pathway analysis showing increased apoptosis in CSN1-KO versus AAVS1 GSCs. NES represents the normalized enrichment score, p-value represents the hypergeometric test and q-value represents the false-discovery corrected p-value (n = 3).

The COP9 signalosome regulates a wide array of cellular processes in a subunit- and tumor-dependent manner [4]. Therefore, we performed RNA-sequencing to identify the specific downstream transcriptional programs altered by CSN1 deficiency in GSCs. Gene set enrichment analysis of these datasets revealed that biological processes and pathways involved in cell cycle progression were negatively enriched in CSN1-KO GSCs relative to AAVS1 (Fig 3A-D). Meanwhile, genes involved in the apoptosis pathway were positively enriched in the CSN1-KO GSCs (Fig 3E). These transcriptional changes confirm that CSN1 is essential for GSC growth *in vitro* and *in vivo*, validating our functional data.

### CSN1-KO disrupts COP9 signalosome composition and alters protein phosphorylation

**Fig 4:**
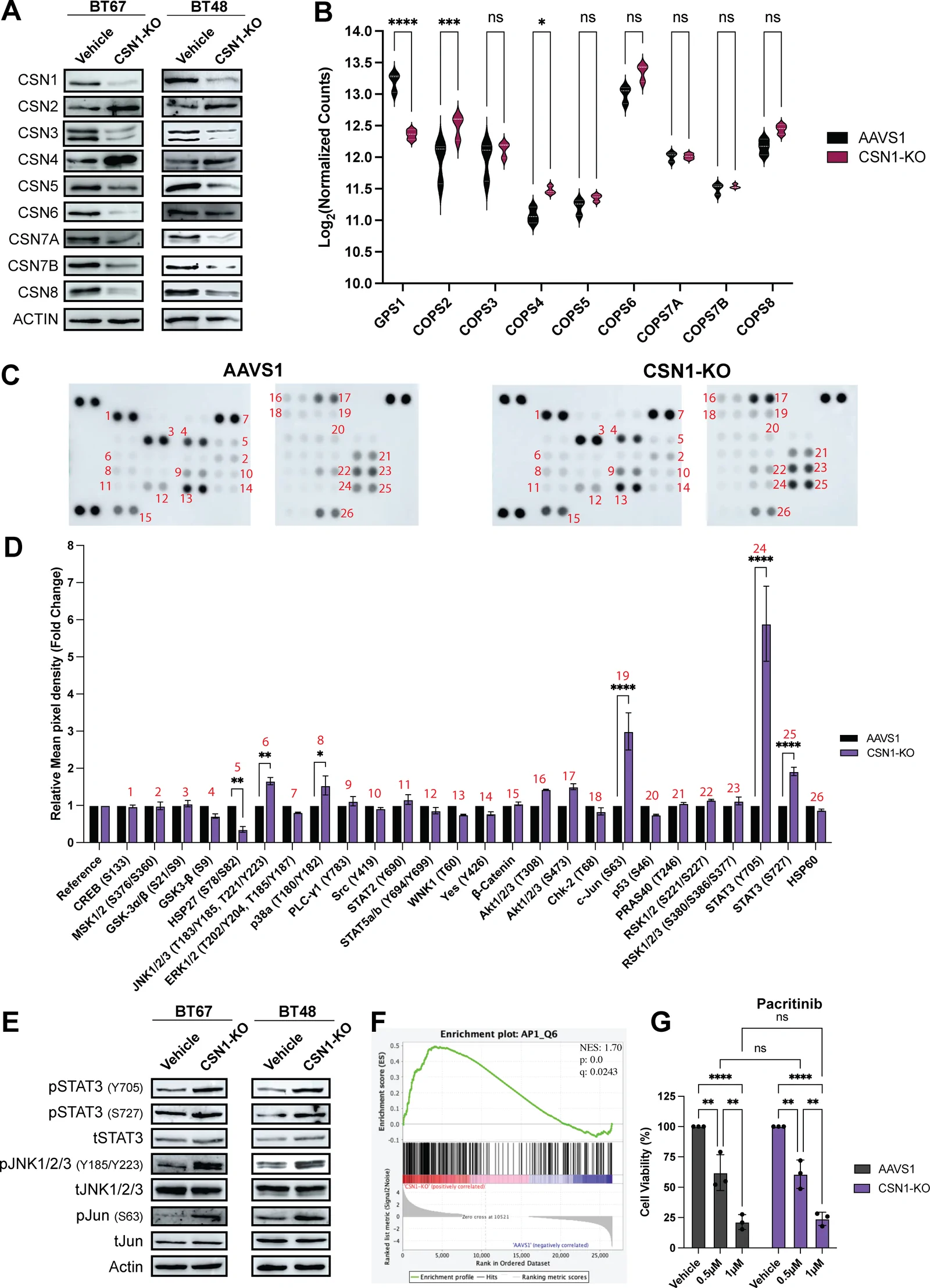
CSN1 is required for COP9 signalosome assembly and regulates the phospho-proteome in GSCs. **(A)** Western blot assessing the protein expression of all COP9 signalosome complex subunits in AAVS1 and CSN1-KO BT67 (n = 3) and BT48 (n = 2) GSCs. **(B)** Log_2_(Normalized RNA-seq counts) of AAVS1 and CSN1-KO BT67 GSCs showing the RNA expression of COP9 signalosome complex subunits. Two-way ANOVA at 95% confidence intervals, ns = not significant, *p < 0.05, ***p < 0.001, ****p < 0.0001; data represented as mean + /- SD, n = 3. **(C)** Dot blot showing levels of 37 phosphoproteins, in horizontal technical duplicates, in AAVS1 and CSN1-KO BT67 GSCs. Red numbers indicate the phospho-protein examined for quantification in (D). **(D)** Quantification of the phospho-kinase array in (B). ANOVA (Tukey’s test) at 95% confidence intervals, *p < 0.05, **p < 0.01, ****p < 0.0001; data represented as mean + /- SD, n = 2. **(E)** Western blot validation of the phospho-kinase array results in (B-C), in AAVS1 and CSN1-KO BT67 (n = 2) and BT48 (n = 2) GSCs. **(F)** GSEA transcription factor target enrichment analysis highlighting AP-1 target enrichment in CSN1-KO versus AAVS1 GSCs (Collection TFT:TFT_LEGACY: TFT_Legacy). NES represents the normalized enrichment score, p-value represents the hypergeometric test and q-value represents the false-discovery corrected p-value (n = 3). **(G)** Viability of AAVS1 and CSN1-KO BT67 GSCs in response to 0.5μM and 1μM of pacritinib. ANOVA (Tukey’s test) at 95% confidence intervals, ns = not significant, **p < 0.01, ****p < 0.0001; data represented as mean + /- SD, n = 3.

Previous studies suggest that all 8 subunits of the COP9 signalosome are required for full enzymatic activity, with additional evidence of subunit-subunit reliance during incorporation in *Arabidopsis thaliana* [10,11]*.* Thus, we sought to identify whether the loss of CSN1 impacts the expression of other COP9 signalosome subunits in GSCs. Intriguingly, we found that CSN1-KO leads to loss of CSN3, CSN5, CSN6, CSN7A, CSN7B, and CSN8, while simultaneously maintaining, or upregulating, CSN2 and CSN4 expression (Fig 4A). Further transcriptomic analysis revealed *GPS1* as the only gene that exhibits significant downregulation in CSN1-KO GSCs (Fig 4B), suggesting that the observed loss of COP9 signalosome subunit protein expression upon CSN1-KO is not due to transcriptional downregulation. These findings thus demonstrate that CSN1 is essential for COP9 signalosome assembly in GSCs, as its loss leads to the destabilization, and subsequent degradation, of most of its components.

CSN1 has been previously shown to interfere with activation of the AP-1 transcription factor by repressing the phosphorylation and/or the overall expression of JNK and c-Jun [12,13]. Furthermore, AP-1 has been linked to the regulation of several pro- or anti-tumorigenic transcriptional programs, depending on several factors such as its dimer composition, cellular context, and genetic background [14]. Therefore, we sought to investigate whether CSN1-KO disrupts the JNK/c-Jun/AP-1 signaling axis in GSCs, as aberrant activation of this pathway may be responsible for the transcriptional alterations observed following CSN1-KO (Fig 3A-E). We utilized a phospho-kinase array kit to assess JNK/c-Jun phosphorylation alongside 35 other phosphorylated proteins, allowing us to additionally determine whether CSN1-KO disrupts any other major signaling pathways in GSCs (Fig 4C). Notably, quantification revealed that CSN1-KO led to significantly increased phosphorylation of STAT3 (Y705 and S727), JNK1/2/3 (T183/Y185, T221/Y223), and c-Jun (S63) (Fig 4D). These phosphorylation changes were subsequently validated in two GSC cultures, which further revealed that the increase in phosphorylation is not linked to upregulation of total protein expression (Fig 4E). As we determined that CSN1-KO leads to JNK and c-Jun phosphorylation, we next sought to determine whether the AP-1 transcription factor played a role in regulating the transcriptional programs we observed in our RNA-seq datasets. Therefore, we performed transcription factor target enrichment analysis and identified that AP-1 transcriptional targets are enriched in CSN1-KO GSCs relative to AAVS1 (Fig 4F), suggesting that its activation may indeed be responsible for a subset of the transcriptional alterations observed upon CSN1-KO.

Phosphorylation of STAT3 in GSCs is typically associated with increased proliferation and survival [15]. Thus, the increased STAT3 activation observed in our CSN1-KO GSCs was intriguing, leading us to hypothesize that CSN1-KO GSCs may rely on this pathway as a compensatory survival mechanism, which would render them more sensitive to STAT3 inhibition compared to AAVS1 controls. To test this, we treated AAVS1 and CSN1-KO GSCs with the JAK2/STAT3 inhibitor pacritinib at IC_50_ and IC_75_ concentrations and assessed viability after 7 days (Fig 4G). However, we did not observe any difference in GSC viability between AAVS1 and CSN1-KO GSCs under either concentration, suggesting that CSN1-KO GSCs do not utilize this pathway to sustain survival.

**Fig 5:**
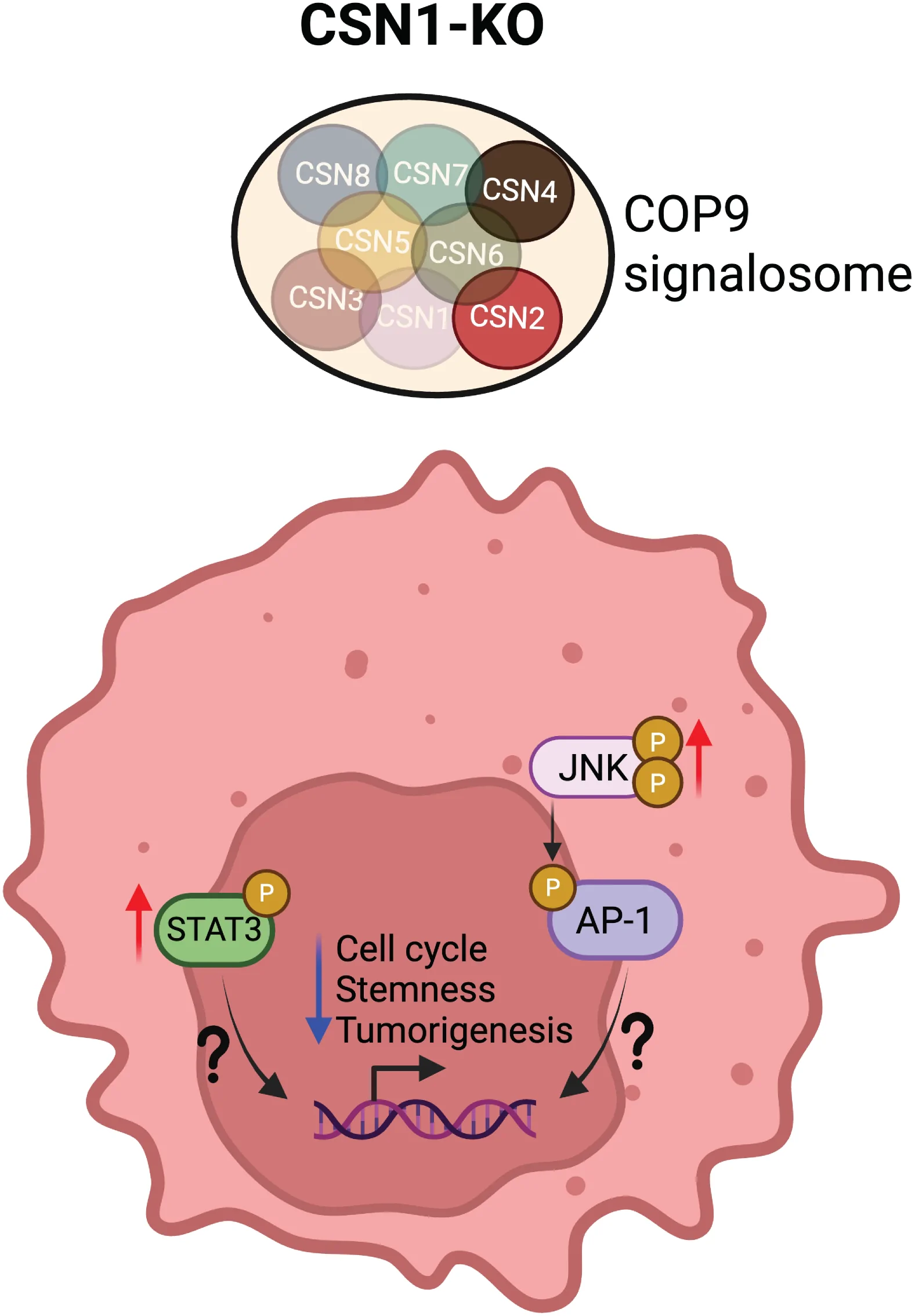
Study model highlighting the role of CSN1 in GSCs. **(A)** Knockout of CSN1 in GSCs disrupts the composition of the COP9 signalosome, promotes the phosphorylation of JNK/AP-1 and STAT3, and activates transcription programs involved in attenuating cell cycle, stemness, and tumorigenesis **(**Created in BioRender. Assaf, S. (2026) https://BioRender.com/g8r8z3r).

Taken together, these results suggest that CSN1-KO disrupts the COP9 signalosome composition, which in turn alters the phospho-proteomic profile of GSCs, notably the phosphorylation of JNK1/2/3 and c-Jun, which may be responsible for regulating the transcriptional programs that stunt GSC stemness and growth *in vitro* and *in vivo* (Fig 5). However, the specific mechanism through which STAT3 and AP-1 are activated, and the downstream programs they regulate, remain unclear.

## Discussion

In this study, we identified that GSCs rely on CSN1 and the COP9 signalosome to regulate their growth *in vitro* and *in vivo.* We demonstrate that CSN1 regulates AP-1 activity and downstream transcriptional programs that govern GSC proliferation. We further identified CSN1 as a crucial regulator for COP9 signalosome assembly and phospho-proteome maintenance in GSCs.

The modular assembly of the COP9 signalosome requires the initial formation of a CSN1/2/3/8 module and a CSN4/5/6/7 module which are subsequently connected by an interaction between CSN1 and CSN6 [10]. Furthermore, integration of the catalytic CSN5 subunit to form the functional complex was shown to be possible in the absence of CSN3 and CSN8; however, loss of CSN1/2/4/6/7 was found to prevent CSN5 integration [16]. In *Arabidopsis thaliana*, each COP9 signalosome subunit appears to be important for the stability of a distinct group of subunits [11]. Loss of function studies revealed that complete CSN5 loss of function led to significant reduction in CSN1, CSN6, CSN7, and CSN8, slight reduction of CSN4, slight accumulation of CSN3, and no effect on CSN2 [11]. CSN6 loss of function led to loss of CSN1, CSN3, CSN4, CSN7, and CSN8, without affecting CSN2 and CSN5 [11]. CSN7 loss of function triggered the instability of CSN1, CSN2, CSN3, CSN4, CSN5, CSN6, and CSN8 [11]. Meanwhile, CSN8 loss of function led to the destabilization of CSN1, CSN3, CSN5, CSN6, and CSN7, without affecting CSN2 and CSN4 [11]. These results further suggested that CSN2 and CSN4 display differential stability depending on which COP9 signalosome subunit is mutated and thus cannot be reliably used as a marker of COP9 signalosome ablation [11]. Therefore, despite the maintained or upregulated expression of CSN2 and CSN4 observed in CSN1-KO GSCs, COP9 signalosome activity is likely completely lost given that all of the remaining subunits, including the catalytic CSN5 subunit, displayed reduced or lost expression. Furthermore, building on the previous studies mentioned above, our results highlight the crucial role of the CSN1 subunit for the assembly and activity of the COP9 signalosome in human GSCs. However, it remains unclear how the stability of CSN2 and CSN4 is differentially regulated and whether they play any independent functional role in the absence of the remaining COP9 signalosome subunits.

Through our phospho-proteome analysis, we identified key proteins that exhibited increased phosphorylation upon CSN1-KO, independent of total protein upregulation, notably STAT3, JNK1/2/3, and c-Jun. However, the increased phosphorylation of STAT3 at Y705 and S727 appears counterintuitive, as activation of the STAT3 pathway has previously been found to promote GSC proliferation and survival [15]. Although we utilized a STAT3 inhibitor and determined that CSN1-KO GSCs did not rely on the STAT3 pathway as a compensatory survival mechanism, the increase in phospho-STAT3 remains an intriguing observation that will need to be investigated in future studies.

In addition to suppressing G protein signaling, CSN1 has been identified to act as a suppressor of the AP-1 pathway by inhibiting JNK and c-Jun activity [12]. These findings align with a previous study showing that CSN1 inhibits the ectopic expression of total JNK1 by interfering with its transcription rather than translation or proteolysis [13]. Our data partially supported these findings as we showed increased JNK1/2/3 and c-Jun phosphorylation upon CSN1-KO, which further correlated with our transcriptomic analysis revealing an enrichment of AP-1 target genes in CSN1-KO GSCs. However, we did not observe any change in the total levels of JNK1/2/3 which, based on previous findings [13], would have been expected to exhibit upregulated total protein expression upon CSN1-KO. This discrepancy may stem from the fact that our GSCs were not manipulated to overexpress JNK1, suggesting that CSN1 primarily plays a role in ensuring adequate JNK1 expression. Furthermore, although our data alongside several others identified CSN1 as an inhibitor of AP-1 activity in diverse contexts [12,13,17], CSN2 and CSN5 were previously shown to promote AP-1 activity [18,19]. Here, we identified that CSN1-KO led to upregulated CSN2 and downregulated CSN5 expression. Therefore, this further suggests that, at least in GSCs, CSN5 may either not be as essential as CSN2 in regulating AP-1 activation or is overcompensated by the upregulation of CSN2. Nonetheless, it is of great interest to determine how AP-1 activation is fine-tuned by distinct COP9 subunits in cells that were not manipulated to overexpress these subunits.

In summary, this study highlights the role of CSN1 in regulating key programs governing GSC stemness and proliferation *in vitro* and *in vivo*, supporting its potential as a therapeutic target for glioblastoma. However, there are some limitations associated with this study. Given the lack of CSN1 inhibitors, the clinical relevance of targeting CSN1 remains limited and will require mechanistic and phenotypic re-evaluation once selective inhibitors are generated. The origin of pSTAT3 elevation and AP-1 signaling activation in CSN1-KO GSCs remains unresolved, and whether their activation directly regulates the observed phenotypic consequences is unclear. Given the importance of these signaling axes in GSCs, the source of pSTAT3 and AP-1 activation and their downstream mechanisms need to be investigated in future studies. Finally, as CSN1-KO GSCs exhibited loss of multiple COP9 signalosome subunits, it remains unclear whether the phenotypic consequences we observed are CSN1-specific or due to the overall loss of the COP9 signalosome. Addressing this will further strengthen the biological and therapeutic significance of CSN1 and the COP9 signalosome in GSCs.

## Methods

### Cell culture

Written informed consent from patients and approval from the Health Research Ethics Board of Alberta – Cancer Committee (REB HREBA-CC-160762) were obtained prior to surgical resection of glioblastoma tumor samples. The patient-derived GSCs were established and cultured as 3D neurospheres in serum-free media supplemented with EGF, bFGF, and heparin sulfate at 37°C, as previously described [20]. The GSCs were first accessed and used for this study on 1/9/2024. All GSCs were authenticated using short tandem repeat profiling and were compared to their parental tumor tissue (Calgary Laboratory Services and Department of Pathology and Laboratory Medicine, University of Calgary). GSCs were routinely tested for mycoplasma using the PCR-based Venor GeM Mycoplasma Detection Kit (Sigma Aldrich #MP0025) and were confirmed to be mycoplasma-free.

**Table:** 

Cell ID	Sex	Diagnosis	IDH	Treatment prior to surgical resection	Mutations	MGMT
BT48	M	Glioblastoma Grade IV	WT	None (Primary glioblastoma)	EGFR	Methylated
BT67	M	Glioblastoma Grade IV	WT	None (Primary glioblastoma)	NF1	Methylated

### Cell viability and growth assay

For cell viability, 1000–2500 GSCs were seeded, per well, in a 96-well plate. Following incubation for 7 days, alamarBlue™ (Invitrogen, cat#DAL1025) was added according to the instruction manual for 4–6 hours prior to measuring fluorescence. For drug testing, GSCs were first incubated for 24 hours prior to drug addition. For cell growth over time assays, 1000 GSCs were seeded per well prior to daily cell viability assessment using alamarBlue™ following an incubation of exactly 6 hours. Readings were recorded until fluorescence readings plateaued.

### Flow cytometry

Cell cycle analysis was performed using the Click-iT Plus EdU Alexa Fluor 647 Flow Cytometry Assay kit (Invitrogen #C10635) as per the manufacturer’s instructions. GSCs were counterstained with propidium iodide (PI) for DNA content using FxCycle PI/Rnase Staining solution (Invitrogen #F10797). Flow cytometry was performed using the CytoFLEX LX (Beckman Coulter) at the Flow cytometry core facility (University of Calgary).

### CRISPR-Cas9 knockout generation

Lentivirus production and CRISPR-Cas9 knockout GSCs were generated as previously described [21], with some modifications. For lentivirus production, 3.7x10^6^ HEK293T cells were transfected with 2.2 μg pMD2.G (Addgene #12259), 4.5 μg pCMVR8.74 (Addgene #22036) and 3.2 μg of Lenti-Cas9-2A-Blast (Addgene #73310) or the gRNA-carrying pLCKO (Addgene #73311) vector using Lipofectamine 2000 (Invitrogen, cat#11668027) according to the manufacturer’s instructions. Media was changed after 24 hours, and the lentivirus-containing media was collected following an additional 24 hours. 5x10^5^ GSCs were then transduced with Lenti-Cas9-2A-Blast (Addgene #73310) [22] for 24 hours followed by a 24 hour recovery and 5 μg/mL blasticidin selection for 10 days. 5x10^5^ Cas9-expressing GSCs were then transduced with pLCKO (Addgene #73311) [22] harboring gRNAs for the *AAVS1* safe-harbor-site which was used as a control or *GPS1.* The cells were allowed to recover for 24 hours followed by a 2 μg/mL puromycin selection for 4 days. All GSC knockout populations were bulk edited. The *GPS1* gRNA sequences were selected from TKOv3 [22] (*AAVS1:*ATCCTGTCCCTAGTGGCCC*; GPS1–1:*GGAGCCCATGCAGATCGACG*; GPS1–2:*TGGCCTCTGAGAGCTTGCGG*; GPS1–3:*ATCAAAGAGAGCATCCGGCG).

### Western blotting

Western blots were performed as previously described [23]. Briefly, Cell pellets that were flash frozen with liquid nitrogen and stored at −80°C were lysed with RIPA buffer (50 mM Tris, 150 mM NaCl, 0.1% sodium dodecyl sulfate (SDS), 0.5% Na Deoxycholate, 1% NP-40) and gently sonicated for 20 minutes for protein extraction. BioRad protein assay was used to quantify protein concentration. 20 μg of protein was loaded onto 7.5%−10% sodium dodecyl sulfate polyacrylamide gel electrophoresis (SDS-PAGE) gels, depending on the size of the protein of interest. Gels were run at 120V and transblotted onto nitrocellulose membranes using the BioRad Transblot Turbo Transfer System (1.0A, 25V, 30 minutes). Western blots were incubated with the primary antibodies at a 1:1000 dilution in blocking buffer (5% nonfat dried milk diluted in TBST supplemented with 0.2% sodium azide) overnight at 4^°^C. Membranes were washed with Tris-buffered saline (TBS) and incubated with the horseradish peroxidase (HRP)-conjugated secondary antibody for 1 hour at room temperature prior to additional TBST washes and imaging. SuperSignal™ West Pico PLUS Chemiluminescent Substrate (ThermoFisher #34580) was added on the membrane prior to imaging using the Amersham Imager 600. CSN1 (Proteintech #11709–1-AP), CSN2 (Thermo Fisher Scientific #PA5–115042), CSN3 (Thermo Fisher Scientific #MA5–34854), CSN4 (Thermo Fisher Scientific #PA5–31125), CSN5 (Thermo Fisher Scientific #MA1–23248), CSN6 (Thermo Fisher Scientific #MA5–26223), CSN7A (Thermo Fisher Scientific #PA5–113421), CSN7B (Thermo Fisher Scientific #PA5–113385), CSN8 (Thermo Fisher Scientific #MA5–52544), pSTAT3 (Y705) (Cell Signaling #9145), pSTAT3 (S727) (Cell Signaling #9134), tSTAT3 (Cell Signaling #9139), pJun (S63) (Cell Signaling #9261), tJun (Abcam #ab31419), pJNK1/2/3 (Y185/Y223) (Abcam #ab76572), tJNK1/2/3 (Abcam #ab179461), SOX2 (Abcam #ab97959), Actin (Sigma Aldrich #A5316). The original representative blots have been deposited at Mendeley Data https://doi.org/10.17632/jyc4j3vkfk.1 (https://data.mendeley.com/datasets/jyc4j3vkfk/1).

### Limiting dilution assay

1024 cells per well were seeded in 6 wells of a 96-well plate, vertically. Serial dilutions were then performed to achieve a final number of 512, 256, 128, 64, 32, 16, 8, 4, 2, and 1 cell per well. GSCs were finally monitored over time for sphere formation and scored as previously described [24].

### Xenografts

Orthotopic xenografts were performed as per our animal ethics protocol (AC21–0162), which is approved by the Animal Care Committee of the University of Calgary and operating under the Guidelines of the Canadian Council on Animal Care. Mice were anaesthetized using 10 mg/kg ketamine/xylazine. Buprenorphine (0.1 mg/kg) was administered once pre-operatively and once post-operatively to minimize animal suffering. 1x10^5^ dissociated and viable AAVS1 or CSN1-KO GSCs were first suspended in 2μl phosphate-buffered saline. 6–8 week old female SCID mice (Charles River) were then randomized prior to stereotactic implantation of either AAVS1 or CSN1-KO GSCs into the striatum of the right cerebral hemisphere; 10 mice were xenografted per group (20 mice total per GSC culture xenografted) to maintain statistical power. Mice were euthanized when experimental endpoints were reached according to animal care guidelines; for this study, the experimental endpoint and the humane endpoint are one and the same. More specifically, mice were monitored and weighed 2–3 times a week for the first 4 weeks after xenograft (known minimum time for tumor development); once animals started displaying early signs of illness, this regimen was increased to 5–7 times weekly (including weekends as needed). Mice were euthanized immediately once they began to show any signs of disease (i.e., 20% weight loss, lack of grooming, inactivity, hind limb paralysis, inability to eat or drink, ataxia, or seizures). Mice were euthanized with a lethal dose of ketamine/xylazine (Ketamine 300–360 mg/kg + xylazine 30–40 mg/kg) followed by deep isoflurane anesthesia + cervical dislocation. No mice died before meeting the humane endpoint. All survival results were included in this study (no mice were excluded). All mouse handling and experimentation were performed by an individual holding training and certifications for mouse husbandry, IAUTP theory, rodent anesthesia (Inhalant and Injectable), cervical dislocation under anesthesia, and surgery for research animals. Log-rank test was used for the Kaplan-Meier survival studies. Confounders were not controlled, as this study only monitored survival following the initial xenografts.

### RNA-sequencing

RNA was extracted from AAVS1 and CSN1-KO GSCs using the RNeasy Kit (Qiagen # 74104) prior to Nanodrop quantification. Strand-specific RNA-sequencing library was constructed from total RNA samples using transcript polyA capture. Sequencing was performed on the Illumina NovaSeq 6000 at the Center for Health Genomics and Informatics (University of Calgary). RNA was aligned to GRCh38 using Rsubread (R version 4.3) and differential expression was performed using DESeq2 [25,26]. Gene set enrichment analysis [27,28] (GSEA v4.4.0) was performed using DESeq2 normalized RNA-seq counts. 1000 gene_set permutations were performed. RNA-sequencing data has been deposited in the NCBI Sequence Read Archive (SRA) database: PRJNA1497041 (https://www.ncbi.nlm.nih.gov/bioproject/PRJNA1497041).

### Phospho-kinase array

AAVS1 and CSN1-KO GSCs were tested for the phosphorylation levels of 37 different phospho-proteins using the Proteome Profiler Human Phospho-Kinase Array Kit (R&D systems #ARY003C) as per the manufacturer’s protocol. Signal intensities were quantified using ImageJ.
